# Significance of the double-layer capacitor effect in polar rubbery dielectrics and exceptionally stable low-voltage high transconductance organic transistors

**DOI:** 10.1038/srep17849

**Published:** 2015-12-14

**Authors:** Chao Wang, Wen-Ya Lee, Desheng Kong, Raphael Pfattner, Guillaume Schweicher, Reina Nakajima, Chien Lu, Jianguo Mei, Tae Hoon Lee, Hung-Chin Wu, Jeffery Lopez, Ying Diao, Xiaodan Gu, Scott Himmelberger, Weijun Niu, James R. Matthews, Mingqian He, Alberto Salleo, Yoshio Nishi, Zhenan Bao

**Affiliations:** 1 Department of Chemical Engineering, Stanford University, Stanford, California 94305, USA; 2 Department of Chemical Engineering and Biotechnology, National Taipei University of Technology, Taipei 106, Taiwan (ROC); 3 Institut de Ciència de Materials de Barcelona (ICMAB-CSIC), and Networking Research Center on Bioengineering, Biomaterials and Nanomedicine (CIBER-BBN), Campus UAB, 08193 Bellaterra, Spain; 4 Department of Electrical Engineering, Stanford University, Stanford, California 94305, USA; 5 Department of Material Sciences & Engineering, Stanford University, Stanford, California 94305, USA; 6 Corning Incorporated, SP-FR-06-1, Corning, NY 14831, USA

## Abstract

Both high gain and transconductance at low operating voltages are essential for practical applications of organic field-effect transistors (OFETs). Here, we describe the significance of the double-layer capacitance effect in polar rubbery dielectrics, even when present in a very low ion concentration and conductivity. We observed that this effect can greatly enhance the OFET transconductance when driven at low voltages. Specifically, when the polar elastomer poly(vinylidene fluoride-co-hexafluoropropylene) (e-PVDF-HFP) was used as the dielectric layer, despite a thickness of several micrometers, we obtained a transconductance per channel width 30 times higher than that measured for the same organic semiconductors fabricated on a semicrystalline PVDF-HFP with a similar thickness. After a series of detailed experimental investigations, we attribute the above observation to the double-layer capacitance effect, even though the ionic conductivity is as low as 10–10 S/cm. Different from previously reported OFETs with double-layer capacitance effects, our devices showed unprecedented high bias-stress stability in air and even in water.

Field-effect transistors (FETs) with both high gain and transconductance are crucial for a broad range of applications^1^,^2^,^3^, including logic circuits, display drivers and sensing^4^,^5^,^6^. High-performance FETs based on organic materials are of particular interest due to their compatibility with low-cost, high-throughput processing and mechanical compliance with soft tissues. However, it has been challenging to realize high transconductance with organic materials due to their relatively low charge carrier mobilities. One effective method is to develop dielectric layers with high capacitances^7^,^8^. Halik *et al.* used an ultra-thin self-assembled monolayer (SAM) dielectric layer^9^ to achieve a high capacitance of 0.7 μF/cm^2^ and a transconductance of 0.01–0.04 S/m in vacuum evaporated OTFTs. Ion-doped polymer electrolytes^10^ and ion gels^11^,^12^ have been used as the dielectric layers for OFETs. Their capacitances are high due to the double-layer capacitor effect. The resulting OFETs have been shown to reach transconductances up to 0.5 S/m. However, challenges remain in using the aforementioned systems for practical applications due to the low yield of SAM fabrication, incompatibility of liquid/gel materials with standard manufacturing processes and the high moisture sensitivity of ionic dielectrics^6^.

## Results

Here, we serendipitously discovered that a polar fluorinated PVDF-HFP elastomer dielectric, despite of a low ion concentration, is able to induce an electric double-layer charging effect under an applied gate voltage. This polymer dielectric is solution-processable with a high static capacitance of ~0.3 μF/cm^2^, even at a thickness of several micrometers. Devices made from this thick polymer dielectric are capable of operating at low voltages with a transconductance as high as 0.02 Sm^−1^ for polymer OTFTs and as high as 1.2 Sm^−1^ for CVD-graphene FET. This polymer dielectric is highly compatible with solution processing of various organic semiconductors. Remarkably, the resulting devices showed both high current output and low bias stress in both ambient and aqueous conditions.

PVDF-HFP polymers are usually semicrystalline when a high molar fraction of PVDF segments are incorporated^13^. However, a higher molar ratio of the HFP units (45mol% determined by ^19^F-NMR shown in Supplementary Information Fig. S2) results in an elastic material with a glass transition temperature (T_g_) of around −20°C (Fig. S1 and S3). Its dielectric constant is 11 as measured at 1 kHz, a value similar to the previously reported range of 8 to 13^14^,^15^.

Thick polymer dielectric films (1.4–5 μm) were used for our OFETs in order to significantly reduce leakage current (10^−6 ^A cm^−2^ at V = −1 V for 1.4 μm) and they are much more readily attainable with large-scale coating methods. Our films displayed smooth surfaces (surface roughness ~0.3 nm) and a high breakdown electric field exceeding 0.3 MV/cm. (Supporting information Figs S4–7) The performance of the e-PVDF-HFP is very stable under different preparation conditions. We tried different conditions of annealing even intentionally adding DI-water to the solution without seeing significant change in the capacitance value.

**Figure 1:**
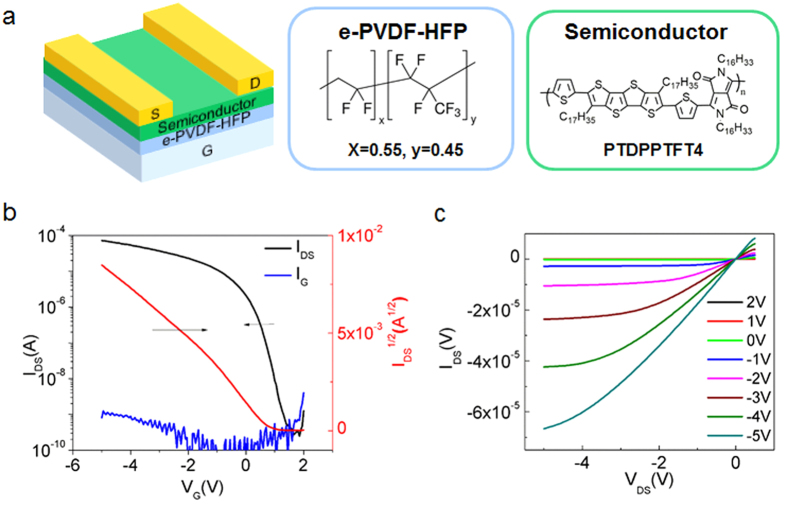
Chemical structure, device structure schematic and characterization of e-PVDF-HFP films. **(a)** Transistor device structure and chemical structures of e-PVDF and PTDPPTFT4. **(b,c)** Output and transfer characteristics of PTDPPTFT4 (channel length L = 50 μm, channel width W = 1000 μm), where V_DS_ = −5 V. The thickness of the dielectric is 1.4 μm. Two slopes in the I_DS_^1/2^ vs. V_G_ plot were observed. This may be attributed to the existence of contact resistance in the device. We used the first slope in the range of +0.2 V to −2 V to estimate mobility values.

To evaluate transistor device performance using this dielectric, we initially chose poly(tetrathienoacene-diketopyrrolopyrrole) (PTDPPTFT4, chemical structure shown in Fig. 1) as the semiconductor layer due to its high charge carrier mobility^16^. The device exhibited a high on-current of close to 10^−4^ A at V_G_ = −5 V, despite the thick dielectric layer (1.4 μm) used. Additionally, the subthreshold slope of the device was only 120 mV decade^−1^, comparable to the lowest values reported for OTFTs^12^,^17^. Furthermore, the threshold voltage (V_TH_) was <1 V, which is highly desirable in low-voltage-driven applications. Most importantly, the PTDPPTFT4 device exhibited a high transconductance per channel width of 0.02 S m^−1^ at a gate voltage of −3 V (Table 1). OTFTs operating at low voltages were previously achieved by using poly(vinylidene fluoride-trifluoroethylene-chlorofluoroethylene) (P(VDF-TrFE-CFE)) as the dielectric layer with a high dielectric constant of up to 60 at low frequencies^17^. However, a thin layer of ~160 nm was required to achieve the high transconductance per channel width of 4 × 10^−3^ S m^−1^ at −3 V. The value achieved with our device is comparable to the best reported OTFTs with ultrathin SAM dielectrics (0.01–0.04 S/m)^18^,^19^, although still lower than the previously reported value with a gel electrolyte dielectric (0.5 S/m)^20^. However, our e-PVDF-HFP dielectric polymer is highly compatible with standard device fabrication processes and results in devices with stable operation in ambient and even under water, as discussed in more detail below.

Even though high transconductance at a low voltage is critical for practical applications, charge carrier mobility is typically used to characterize the charge transport ability of a semiconductor. The charge carrier mobility is calculated from the standard MOSFET models from the saturation and linear regimes and is dependent on the capacitance value used for the calculation. Non-ionic dielectric materials exhibit relatively constant capacitances regardless of the measurement frequency. The capacitance of e-PVDF-HFP was also found to remain almost unchanged from 20 to 100 kHz, but it increased rapidly as the frequency was decreased to below 1Hz. Therefore, the typical procedure widely used in literature for mobility calculation using a capacitance value measured at ≥20 Hz would result in an overestimation of mobility. This issue was further confirmed by measuring transistors with e-PVDF-HFP of various thicknesses. Even though the capacitances measured at 20 Hz scaled with dielectric thickness as expected for standard capacitors (Fig. S4), the transistor output current did not show the expected scaling with dielectric thickness. It suggests the origin of the high transconductance is likely distinctive from those prepared on other PVDF based dielectric materials primarily utilizing their high polarizability. We further measured the capacitance in the quasi-static limit. A sharp rise in the capacitance value is observed at low frequencies approaching the quasi-static limit. Furthermore, the capacitance showed little change as a function of the thickness of the dielectric layer (Fig. S4a), suggesting a double-layer charging effect was present in our system. Our observed high capacitance also explains the high transconductance obtained even at low operating voltage.

**Figure 2:**
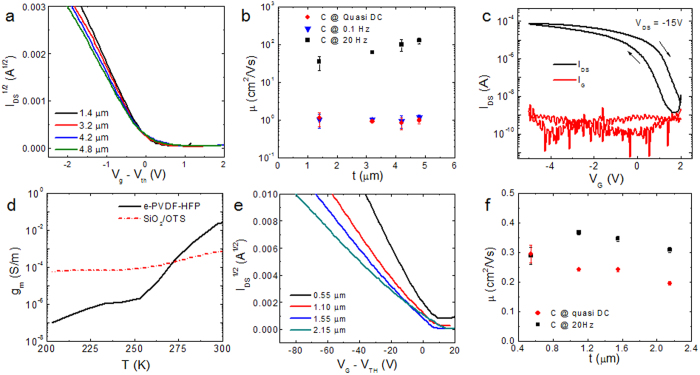
Device characteristics of PTDPPTFT4 transistors fabricated on e-PVDF-HFP and c-PVDF-HFP. **(a)** Transfer curves of OTFTs prepared on e-PVDF-HFP dielectric layer with varying thicknesses. **(b)** Corresponding field-effect mobility as a function of the thickness of e-PVDF-HFP determined by using capacitance at 20 Hz, 0.1 Hz and quasi-DC values. The adoption of capacitance at 20 Hz gives rise to underestimation of the actual charge carrier density during transfer characteristic measurements and therefore inflated mobility values. **(c)** Device characteristics in forward and reverse sweep. **(d)** Temperature-dependent transconductance of PTDPPTFT4 FETs made on e-PVDF-HFP (solid trace) and OTS-modified SiO_2_ (dotted trace). **(e)** Transfer curves of OTFTs prepared on c-PVDF-HFP dielectric layer with varying thicknesses. **(f)** Corresponding field-effect mobility as a function of the thickness of c-PVDF-HFP determined by using capacitance at 20 Hz and quasi-DC values.

To further confirm the capacitance value in the quasi-static limit, we measured the time constant of an RC circuit, based on an e-PVDF-HFP capacitor and an external resistor (Fig. S8). Interestingly, no voltage-dependence of the capacitance was observed. This confirms the high capacitance at a low frequency of 0.1 Hz, which allows explicit determination of the capacitance (Fig. S4) and the corresponding mobility (Fig. 2b) from OTFT measurements. The double-layer charging effect at low frequency for e-PVDF-HFP is surprising considering that <1 wt% of salt is present. In comparison, typical ion-gel dielectrics consist of >80 wt% ionic liquid and a high ion conductivity in the order of 10^−2^ − 10^−5 ^S/cm^21^,^22^,^23^. The measured ion conductivity for our e-PVDF-HFP is several orders of magnitude lower, in which we measured it to be ~8 × 10^−11^ S/cm (Fig. S7). In contrast, when a semi-crystalline PVDF-HFP (90% of VDF by molar ratio, termed as c-PVDF-HFP) was used as a dielectric layer, the output current decreased with increasing c-PVDF-HFP thickness from 0.55 μm to 2.15 μm (Fig. S4b), as expected for conventional dielectric materials. The quasi-static capacitance through charging/discharging of an RC circuit also yielded similar capacitance values to those measured at higher frequencies (>20 Hz).

## Discussion

The above observations suggest that the unusual double-layer charging effect in the e-PVDF-HFP is related to its low T_g_ (~−20 °C). Indeed, it is well known that elastic polymers are desirable matrixes for ion conductors due to the high segmental motion that facilitates ion transport^24^. Furthermore, the high concentration of polar groups in e-PVDF-HFP is important to solvate any salt impurities, such as crosslinking reagents (typically phosphonium compounds)^25^,^26^,^27^. In the case of c-PVDF-HFP, no clear evidence of ion contribution to the charging process was identified because of the suppressed segmental motion due to its semicrystalline nature. Poly(dimethylsiloxane) (PDMS) is a well-known elastic dielectric material. However, it does not exhibit signatures of electric double-layer charging due its low polarity (Figs S4c and S8). Therefore, e-PVDF-HFP is a rare dielectric material that exhibits the double-layer charging effect of ionic dielectrics, while also maintaining the processing characteristics and stability of non-ionic dielectrics.

The combination of the fluorinated polar elastomer with a low concentration of ions in e-PVDF-HFP dielectric gated OTFTs induces a high charge carrier density through electric double-layer charging. Furthermore, all of our studied transistors showed a small hysteresis of ~0.5 V, with a higher back-sweep current compared to the forward sweep (Fig. 2c). The performance of the device depends on the operating temperature, with a sharp decrease in the transconductance by cooling the device from room temperature to 200 K (Fig. 2d). This decrease is largely attributed to the reduced ion mobility, which directly correlates with the segmental motion and depends strongly on temperature^24^.

**Figure 3:**
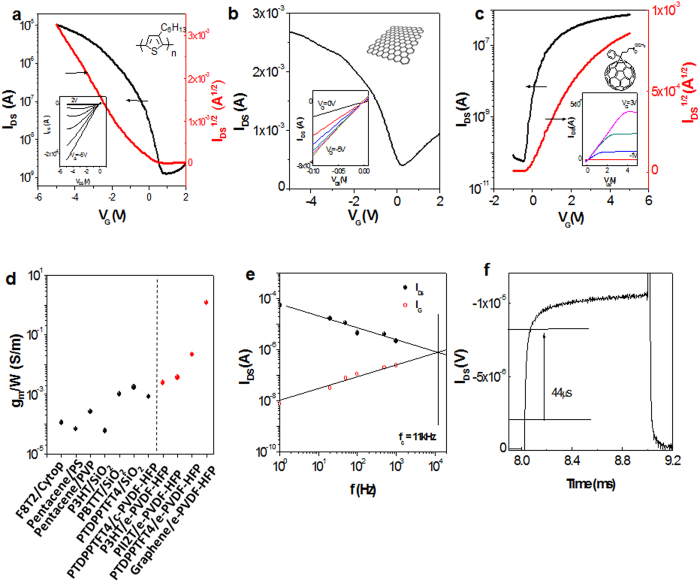
Electrical Characteristics of OTFTs based on e-PVDF-HFP dielectric layer with W/L = 20. Transfer and output characteristics of the OTFTs of **(a)** P3HT, **(b)** graphene, and **(c)** PCBM, respectively. Note that the transfer characteristics of the graphene devices were evaluated in the linear-regime, where V_DS_ = −0.1 V. Each panel exhibits the transfer curves with output characteristics shown in the inserted small figures. **(d)** Transconductance comparison between different dielectrics. All the transconductances were normalized to a driven gate voltage of −3 V. The black dots show the transconductance obtained from OFETs based on common dielectric layers, including cross-linked Cytop (d = 50–70 nm)^34^, cross-linked polystyrene (PS) (d = 10 nm)^35^, cross-linked poly(vinyl phenyl) (PVP) (thickness = 280 nm)^36^, SiO_2_ (d = 230–300 nm)^37^,^38^ and semicrystalline PVDF-HFP (thickness = 1.4 μm)^39^. Note that all SiO_2_ dielectrics were modified by OTS SAMs. The red dots represent the performance obtained from OFETs based on e-PVDF-HFP. The transconductances of all the e-PVDF-HFP devices are around one order of magnitude higher than the corresponding devices made on OTS-modified SiO_2_. **(e)** I_DS_ and I_G_ currents versus frequency of a PTDPPTFT4 transistor with e-PVDF-HFP as a dielectric layer (L = 50 μm, W = 1000 μm), where V_DS_ = −15 V, V_G_ = 10 V to −10 V. The cut-off frequency (f_c_) was estimated as the intersection of I_DS_ and I_G_. **(f)** I_DS_ response of PTDPPTFT4 to a square wavefunction gate-voltage pulse at 1 kHz, pulse width = 1 ms, pulse rise time = 4 μs, and channel length = 50 μm. The response time is defined as the time required to reach 80% of the maximum ON-current from 20% OFF.

We tested several additional well-known solution processable organic semiconductors and CVD-graphene on the thick e-PVDF-HFP dielectric. As shown in Fig. 3, both p- and n-channel transistors exhibited high transconductance. All the devices generate large current outputs at low gate voltages of less than 5V. The transconductances per channel width of these devices were measured to be three to ten times higher than the values of the corresponding material with SiO_2_ or other commonly reported polymer dielectrics. The CVD-graphene device showed a transconductance as high as 1.2 mS (V_DS_ = −0.1 V), and it is even higher than CVD-graphene devices using phosphate-buffered electrolyte with NaCl as a dielectric layer (0.42 mS). By normalizing V_D_s for comparison purposes, the normalized transconductance (g_m_/V_D_) of our graphene device (12000 *μ*SV^−1^) is higher than those of the graphene devices made on high-dielectric-constant HfO_2_ or Y_2_O_3_ dielectrics (~100 *μ*SV^−1^).

Slow response time is a potential concern for ionic dielectric gated field-effect transistors. For practical applications, such as radio frequency identification (RFID) and organic light-emitting diodes (OLEDs), switching speed is one of the most critical device parameters. The switching speed of the ionic electrolyte gated devices typically ranges between 1 to 100 Hz. Previously, Frisbie *et al.* demonstrated 10 KHz operation with ion gels at a very high ion concentration (9 wt% ionic liquid, 0.7 wt% polymer electrolyte and 90 wt% solvent) and high ion mobility (around 8 × 10^−3 ^S/cm)^11^. To evaluate the switching behavior of our e-PVDF-HFP OFETs, we applied a short gate voltage pulse for device operation. The e-PVDF-HFP/PTDPPTFT4 transistor exhibited a switching-on response of 44 μs (Fig. 3e,f). The cutoff switching frequency (f_c_), characterizing the maximum operating frequency of a transistor, was determined to be 11 kHz by measuring the I_DS_ and I_G_ as a function of frequency, in which f_c_ is defined as the frequency where AC modulated I_DS_ is equal to the parasitic gate current (I_G_). Intriguingly, the f_c_ value (11 kHz) is comparable or even higher than many polymer electrolyte devices with very high ion concentrations^11^, despite the ultralow ion concentration in e-PVDF-HFP. The lowering of I_DS_ observed at higher frequencies (Fig. 3e) is attributed to the decreased capacitance with increased frequency. In addition, the increased I_G_ is mainly attributed to parasitic current contributed from the large overlap between the drain/source and gate electrodes. The cutoff frequency of our device current device design is currently mainly limited by its long channel length (L = 50 μm) and large overlap between drain/source and gate electrodes, which results in a large parasitic gate current. The switching speed of devices can be further improved by minimizing the overlap of the drain/source and gate electrodes and further modification of the dielectric material. In a complementary experiment, drain bias consisting of an AC signal superimposed to a DC voltage has been applied while measuring the transfer characteristics, allowing the calculation of linear field-effect mobility. As expected, the DC-mobility of our device in DC mode does not change, while the AC-mobility only drops to about 20% and 50% at frequencies of 1 kHz and 10 kHz, respectively (Figure S14). Even though the device architecture has not been optimized for high frequency, these results are in agreement with the pulsed gate measurement, and highlight the suitability of employing these devices in novel biosensor applications.

**Figure 4:**
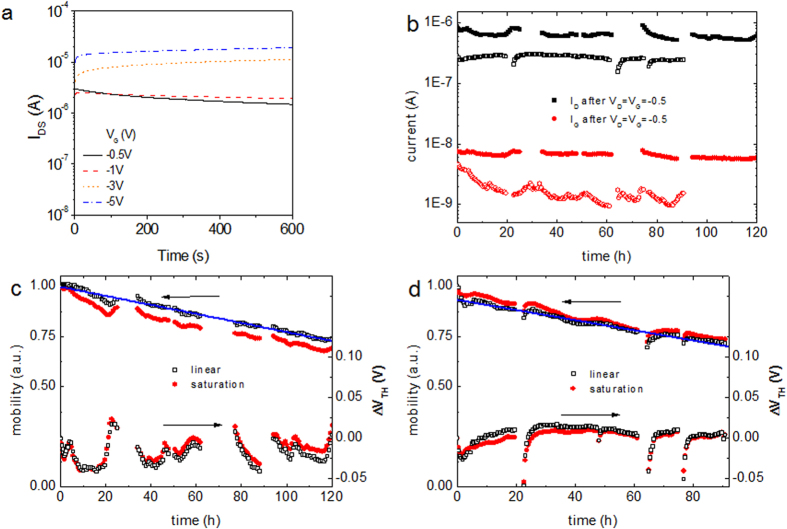
Bias stress analysis of e-PVDF-HFP devices with PTDPPTFT4 as the semiconductor material. **(a)** Bias stress behavior (I_DS_ vs. time) for the PTDPPTFT4 FETs under V_G_ = −0.5, −1, −3 and −5 V in ambient conditions. **(b)** Long-term bias on current I_D_ and leakage current I_G_ measured at the end of each bias cycle in air (solid symbols) and in DI-water (open symbols). A bias of V_D_ = V_G_ = −0.5 V was applied and transfer characteristics were measured before and immediately after each bias step. **(c,d)** Evolution of mobility, and shift of threshold voltage in both the linear and saturation regimes during long-term bias in air and DI-water, respectively. The field effect mobility was calculated with the quasi-static capacitance. The decrease of mobility was analyzed by performing a linear regression in time exhibiting a slope of about −0.22%/hour and −0.25%/hour, for the device in air and DI-water, respectively. Interruptions in the plots are the start of a new measurement cycle and refilling the syringe pump in case of the device exposed to DI-water.

Another important issue with OFETs is the bias stress and device stability over time^28^. Typical double-layer charging capacitor based transistors are highly sensitive to humidity. Additionally, the large number of ions present in the dielectric can diffuse into the semiconductor material and result in redox reactions and material degradation^29^. For this purpose e-PVDF-HFP-PTDPPTFT4 devices were analyzed in detail, employing a bias period of 10 minutes at different gate voltages under ambient conditions (Fig. 4a). Previous bias stress analysis in FETs lasting for hours typically reported threshold voltage shifts of at least several volts, even with fluorinated dielectrics^30^,^31^. Low-voltage transistors are the most stable regarding threshold voltage shifts but still show shifts of about one volt after 27 h of bias^32^. To obtain information about long-term stress effects in ePVDF-HFP based devices, a bias of V_D_ = V_G_ = −0.5 V was applied and transfer characteristics were measured before and immediately after each bias step (30 mins duration). This measurement was repeated continuously for over 120 h. The devices exhibited highly stable on (I_D_) and leakage (I_G_) currents (Fig. 4b). Interestingly, the threshold voltage showed a small variation of less than ±25 mV and, importantly no drift even after 120 h. A similar device placed under DI-water for over 90 h also showed little bias stress, stable I_D_ and only slight decrease in leakage currents. Additionally, the stability of the e-PVDF-HFP dielectric was tested with devices stored under ambient conditions for more than three months, as well as devices immersed in DI-water for over 24 h, both showing negligible variation in the capacitance values. This is the first example of an OTFT driven under continuous bias with the active material directly exposed to water to exhibit such small variations of V_TH_ and current output. The unprecedented device stability with direct exposure to both air and water is directly related to the high ambient and water stability of the e-PVDF-HFP dielectric, as well as the high stability of the PTDPPTFT4 semiconductor. These results indicate that e-PVDF-HFP provides the advantages of typical double-layer charging dielectrics, i.e. low voltage operation and high transconductance, while maintaining an unprecedented device stability and low leakage current. This makes it especially useful for applications requiring high current output and sensor applications.

## Conclusion

We have successfully demonstrated that the polar rubbery dielectric material e-PVDF-HFP significantly enhances the transconductance of OTFTs at low operating voltages, despite the employed dielectric layer being more than one micron thick. The high OTFT performance is attributed to the formation of an electric double layer in the dielectric material, a phenomenon rarely observed in polymer dielectrics at low ion concentrations. We provided clear evidence that there is significant influence of electric double-layer charging on OTFT transfer characteristics, even at an extremely low ion conductivity (8 × 10^−11^ S/cm), a value that is several orders of magnitude lower than conventional polymer electrolytes (10^−4^ − 10^−5^ S/cm)^21^ or ion liquids/gels (10^−2^ − 10^−4^ S/cm)^22^,^23^. In addition, our results demonstrate the importance of verifying the capacitance in the quasi-static limit in order to correctly characterize the charge carrier mobility. The combination of the high polarity and low *T*_*g*_ of our elastic fluoro-polymer results in a double-layer capacitor effect, thus leading to the high transconductance observed in our devices. Importantly, this dielectric material should be generally applicable to a variety of semiconducting materials beyond organic semiconductors. Due to its low cost, compatibility with standard manufacturing techniques, low driving voltage and high stability in air and aqueous media, polar rubbery polymer dielectrics should prove valuable in practical applications such as biomedical devices, sensors, wearable electronics and stretchable devices.

## Methods

Polymer semiconductors, P3HT (from Aldrich), PTDPPTFT4 (provided by Corning Incorporated) and PCBM (from Sigma-Aldrich) were used as received without further purification. PII2T was synthesized according to previously reported procedures^33^. e-PVDF-HFP was purchased from 3M Co (3M™ Dyneon™ Fluoroelastomer FE). It (1.2 g) was dissolved in 10 mL anhydrous 2-butone by stirring overnight under an inert atmosphere. The obtained solution was filtered through a 0.2 μm PTFE filter and spin-coated onto a highly doped *n*-type Si (100) (<0.004 Ω cm) substrate at 1500 rpm for 1 min. The films were then dried at 80 °C for 10 min and subsequently cross-linked at 180 °C for 6 hours.

The semiconducting polymers and PCBM were spin-coated on top of the fluoroelastomer from chlorobenzene (P3HT, 5 mg/mL), dichlorobenzene (PII2T, 5 mg/mL), chlorobenzene (PTDPPTFT4, 5 mg/mL) and chloroform (PCBM, 10 mg/mL), respectively, at 1000 rpm for 1 min. They were then annealed for 1h at 120 °C under an inert atmosphere to remove any residual solvent. The monolayer graphene films were grown on Cu foil using chemical vapor deposition. Sequentially, the CVD-grown graphene sheets were transferred onto the e-PVDF-HFP/Si substrate for device fabrication. Gold source-drain contacts were then evaporated through a shadow mask on top of the semiconducting thin films (top contact).

The TFT transfer and output characteristics were recorded in a N_2_-filled glove box or in air by using a Keithley 4200 semiconductor parametric analyzer (Keithley Instruments, Cleveland, OH). The capacitances of the dielectric materials were measured using an Agilent E4980A Precision LCR Meter and a Biologic VMP3 electrochemistry workstation. The Quasi-DC capacitance measurements based on charging/discharging of RC circuits were carried out adding an external resistor, applying a Keithley model 2400 as voltage source and a Keithley model 2635A as voltmeter.

Optical micrographs were recorded with a cross-polarized optical microscope (Leica DM4000M). Thickness measurements were performed on a Dektak 150 profilometer (Veeco Metrology Group). Tapping mode atomic force microscopy was performed using a Multimode Nanoscope III (Digital Instruments/Veeco Metrology Group). Differential scanning calorimetry was measured on a TA Instruments Q2000. Grazing incidence X-ray diffraction (GIXD) experiments were performed at the Stanford Synchrotron Radiation Lightsource (SSRL) on beamline 11-3 with a photon energy of 12.7 keV. A 2D image plate (MAR345) was used to detect the diffracted X-rays. The detector was 400 mm from the sample center. The angle of incidence was kept at 0.08 degrees, slightly below the critical angle corresponding to total reflectance to reduce the scattering background from the amorphous dielectric beneath the active layer. At an incident angle of 0.12 degrees, the diffraction peaks of the active layer were drown by the background scattering, whereas at incident angles below 0.08 degrees, the signal from the active layer became weaker. The exposure time was 6 min. The GIXD data was analyzed using the wxDiff software.

**Full Methods** and any associated references are available in the online version of the paper at www.nature.com/nature.

## Additional Information

**How to cite this article**: Wang, C. *et al.* Significance of the double-layer capacitor effect in polar rubbery dielectrics and exceptionally stable low-voltage high transconductance organic transistors. *Sci. Rep.* **5**, 17849; doi: 10.1038/srep17849 (2015).

## Supplementary Material

Supplementary Information

## Figures and Tables

**Table 1:** Summary of the OFET electrical performances measured in the saturation regime using e-PVDF-HFP dielectric layer (thickness 1.4 μm).

Sample	mobility^MAX^	Mobility^ave^	on/off^ave^	V_t_^ave^ (V)	g_m_^ave^ (μS)	g_m_^ave^/W (S/m)	g_m_^avg^/V_bias_,(μS/V)^2^
	(cm^2^V^−1^s^−1^)	(cm^2^V^−1^s^−1^)					
P3HT	0.14	0.09 ± 0.05	4 × 10^3^	0.01 ± 0.59	3.48	3.48 × 10^−3^	1.16
	(4.86)*	(3.21 ± 1.64)					
PII2T	0.19	0.17 ± 0.02	8 × 10^3^	−0.55 ± 0.08	3.42	3.42 × 10^−3^	1.14
	(7.35)	(3.52 ± 1.78)					
PTDPPTFT4	2.11	1.09 ± 0.44	2 × 10^4^	−0.67 ± 0.31	26.4	26.4 × 10^−3^	8.8
	(75.71)	(38.98 ± 15.77)					
Graphene	2181	1.32 ± 0.99 × 10^3^	6	1.08 ± 0.56	1200	1.2	1.2 × 10^4^
	(7.11 × 10^4^)	(3.95 ± 2.97) × 10^4^					
PCBM	*0.03*	0.02 ± 0.003	6 × 10^3^	−2.1 ± 0.4	0.54	5.4 × 10^−4^	0.18
	(1.07)	(0.82 ± 0.16)					

^1^The mobilities in parentheses are extracted from LCR Meter-measured capacitance (8.4 nF cm^−2^) at 20 Hz.

^2^g_m_/V_bias_ is normalized with respect to voltage bias. For P3HT, PII2T and PTDPPTFT4 operated in the saturation regime, the value is normalized with respect to the gate voltage (3 V). For graphene operated in the linear regime, the value is normalized with respect to the drain-source voltage (V_DS_ = −0.1 V).

Mobility values were calculated in quasi-static capacitance of 300 nF cm^−2^. (W: 1000 μm; L = 50 μm).
