# Supplementary material for: Significance of the double-layer capacitor effect in polar rubbery dielectrics and exceptionally stable low-voltage high transconductance organic transistors
Source: Sci Rep. 2015 Dec 14;5:17849. doi: 10.1038/srep17849 (PMC4677320; doi:10.1038/srep17849)
Supplement: Supplementary Information [file srep17849-s1.pdf]

Supplementary Information

Significance of the double-layer capacitor effect in polar rubbery dielectrics and exceptionally stable low-voltage high transconductance organic transistors

Chao Wang[1]†, Wen-Ya Lee[1,2]†, Desheng Kong[1]†, Raphael Pfattner[1,3], Guillaume Schweicher[1], Reina Nakajima[1], Chien Lu[1], Jianguo Mei[1], Tae-Hoon Lee[4], Hung-Chin Wu[1], Jeffery Lopez[1], Ying Diao[1], Xiaodan Gu[1], Scott Himmelberger[5], Weijun Niu[6], James R. Matthews[6], Mingqian He[6], Alberto Salleo[5], Yoshio Nishi[4], Zhenan Bao[1*]

[1] Department of Chemical Engineering, Stanford University, Stanford, California 94305, USA.

[2] Department of Chemical Engineering and Biotechnology, National Taipei University of Technology, Taipei 106, Taiwan (ROC).

[3] Institut de Ciència de Materials de Barcelona (ICMAB-CSIC), and Networking Research Center on Bioengineering, Biomaterials and Nanomedicine (CIBER-BBN), Campus UAB, 08193 Bellaterra, Spain.

[4] Department of Electrical Engineering, Stanford University, Stanford, California 94305, USA.

[5] Department of Material Sciences & Engineering, Stanford University, Stanford, California 94305, USA.

[6] Corning Incorporated, SP-FR-06-1, Corning, NY 14831, USA

†These authors contribute equally to the paper

*Correspondence to: zbao@stanford.edu.

Full Methods

Preparation of the dielectric layer. Si wafers, used as substrates, were cleaned by

using UV-ozone for 20min. e-PVDF-HFP (1.2 g) was dissolved in 10 mL of anhydrous 2-butone by stirring overnight in a nitrogen glovebox. The obtained solution was filtered by passing the solution through 0.2 μm PTFE filters and then spin-coated on the wafers at 1500 rpm for 1 min. The spin-coated films were then dried at 80 ℃ for 10 min and subsequently cross-linked at 180 ℃ for 6 hours.

Dielectric and semiconducting thin films characterization. Optical micrographs were recorded with a cross-polarized optical microscope (Leica DM4000M). Thickness measurements were performed with a Dektak 150 profilometer (Veeco Metrology Group). Tapping mode atomic force microscopy was performed using a Multimode Nanoscope III (Digital Instruments/Veeco Metrology Group). Differential scanning calorimetry was realized on a TA Instruments Q2000. Grazing incidence X-ray diffraction (GIXD) experiments were performed at the Stanford Synchrotron Radiation Lightsource (SSRL) on beamline 11-3 with a photon energy of 12.7 keV. A 2D image plate (MAR345) was used to detect the diffracted X-rays. The detector was 400 mm from the sample center. The angle of incidence was kept at 0.08 degrees, slightly below the critical angle corresponding to total reflectance to reduce the scattering background from the amorphous dielectric beneath the active layer. At an incident angle of 0.12 degrees, the diffraction peaks of the active layer were drown by the background scattering, whereas at incident angles below 0.08 degrees, the signal from the active layer became weaker. The exposure time was 6 min. The GIXD data was analyzed using the wxDiff software.

Capacitance measurement. Capacitance-voltage characteristics were determined using metal-insulator-metal (MIM) and metal-insulator-semiconductor (MIS) structures. In the MIM structure, heavily doped Si (<0.004 Ω cm) and Al (100 nm) were used as bottom and top electrodes, respectively, and 1.4-μm-thick PVDF-HFP was employed as an insulating layer. Electrode areas were in the range of 0.02 to 0.12 cm^2. In the MIS structure, a semiconductor layer was deposited on an e-PVDF-HFP/doped Si substrate using the above described device fabrication conditions. Au (40 nm) was

deposited on the semiconductor layer as a top electrode. The capacitance of the dielectric was measured with an Agilent E4980A Precision LCR and a Bio-Logic VMP3 electrochemical workstation.

Frequency measurement of mobility. Current voltage characteristics have been measured on common gate devices with 200 μm channel length using an Agilent E4980A Precision LCR meter equipped with an additional voltage source for the gate. While the gate voltage has been swept, an AC voltage of 100mV superimposed to a constant drain bias of -200mV has been applied. This setup allows applying both gate and drain voltages while measuring the drain current, *i.e.* transfer characteristics, both in AC and DC.

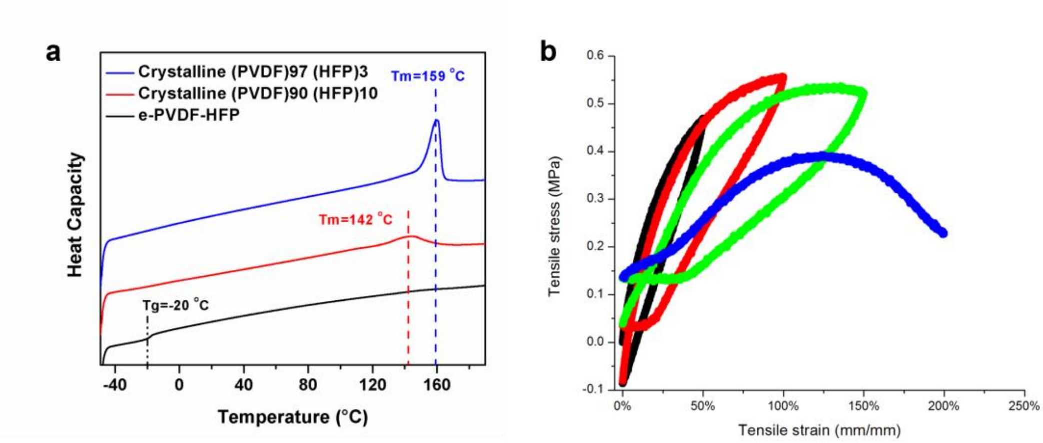

Figure S1. Physical properties of e-PVDF-HFP. (a) DSC traces of the e-PVDF-HFP and semicrystalline PVDF-HFP polymers. The lack of the melting peak, characteristic of a semicrystalline polymer, clearly indicates the formation of an amorphous material when increasing the molar percent of HFP to 45%. The percentage of VDF and HFP in e-PVDF-HFP was calculated from the known total fluorine weight percentage (65.9%). **(b)** Stress-strain cycling of tests of e-PVDF-HFP. e-PVDF-HFP shows elastic behavior up to 50% strain and maintains some elasticity up to 100% strain. Significant plastic deformations began to occur beyond 100% strain and resulted in fracture of the material around 150% strain.

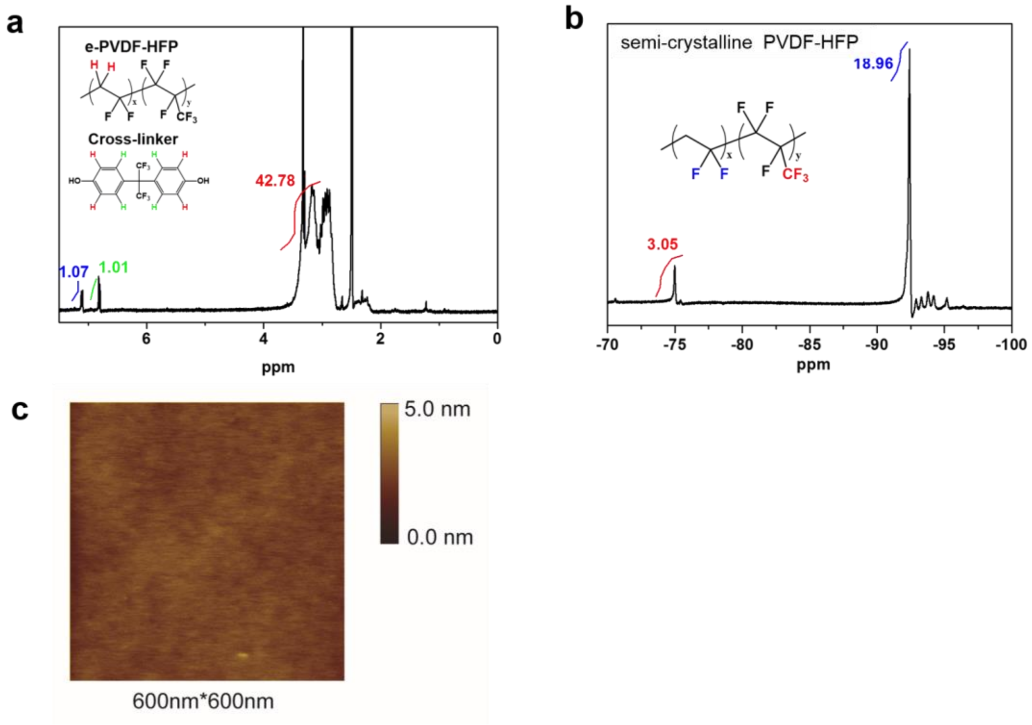

Figure S2. NMR spectra of the e-PVDF-HFP polymer. (a) ^{1}H NMR of the e-PVDF-HFP. The percentage of the cross-linker was calculated through the integration of the protons on VDF (red) and the cross-linker (blue and green). The amount of cross-linker was calculated to be around 1mol%. **(b)** ^{19}F NMR of the semi-crystalline PVDF-HFP pallet, the molar percentage of the HFP component was calculated to be 10%. **(c)** AFM image of the surface morphology of e-PVDF-HFP film with a thickness of 1.4 μm.

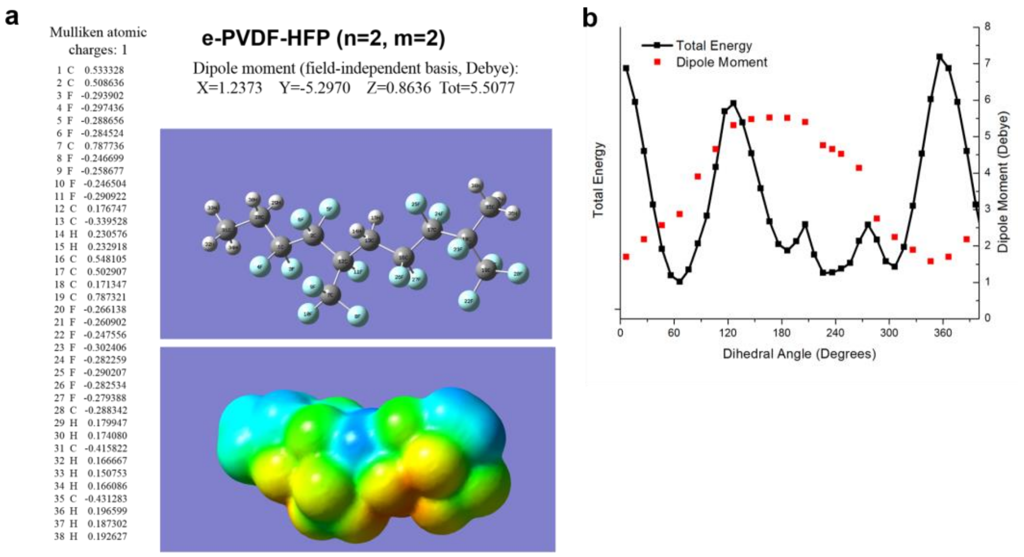

Figure S3 Calculation of dipole moment in VDF-HFP units. (a) DFT hybrid functional calculations of the charge density distribution and optimized geometry of e-PVDF-HFP. Calculations of the charge density distribution, total dipole moment and optimized geometry of PVDF-HFP have been carried out using a B3LYP/6-31G(d) basis set DFT hybrid functional with Gaussian-09 software. From the calculations, it is apparent that there are large polarizations along the PVDF-HFP backbone. This leads to distinct local dipole moments on the polymer chain with the most polarized regions corresponding to the junctions between the VDF and HFP monomers. **(b)** The energy of the molecule was calculated as a function of the dihedral angle between atoms labeled 2C and 16C in the figure above (rotation along the 12C¬–13C bond). The total dipole moment and structure energy calculations were carried out using a B3LYP/6-31G(d) basis set DFT hybrid functional with Gaussian-09 software. The optimized geometry of PVDF-HFP shown above was used as a starting point for this coordinate scan. From this, we can see that strong local dipoles are present at the VDF/HFP monomer intersections at most favorable rotations of the VDF-HFP bond.

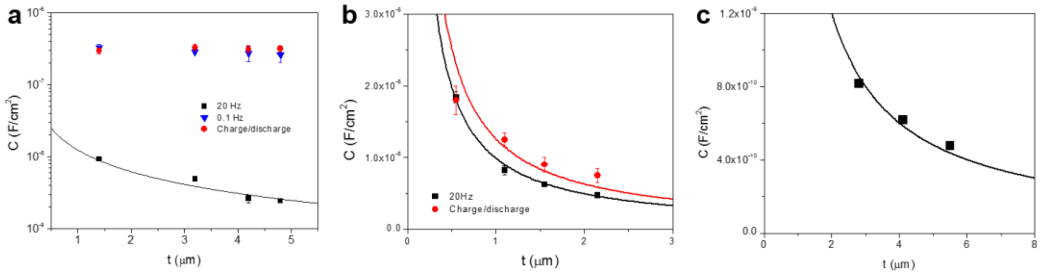

Figure S4. **The dependence of the capacitance as a function of the dielectric layer thickness for e-PVDF-HFP, c-PVDF-HFP and PDMS. (a)** The capacitance values of e-PVDF-HFP at 20 Hz, 0.1 Hz and quasi-DC capacitance determined by charging/discharging of an RC circuit. The capacitance values at 20 Hz are inversely proportional to the thickness of the dielectric layer, which represents the regular charging process of the dielectric layer. The values at very low frequency of 0.1 Hz, which approaches the DC limit, do not exhibit dependence on the dielectric layer thickness due to the electric double layer charging process. The results are consistent with the quasi-DC capacitance based on charging/discharging of an RC circuit. **(b)** The capacitance of c-PVDF-HFP at 20 Hz exhibits a clear trend to be inversely proportional to its thickness, which is very close to the quasi-DC capacitance. **(c)** The capacitance of PDMS at 20 Hz is inversely proportional to its thickness.

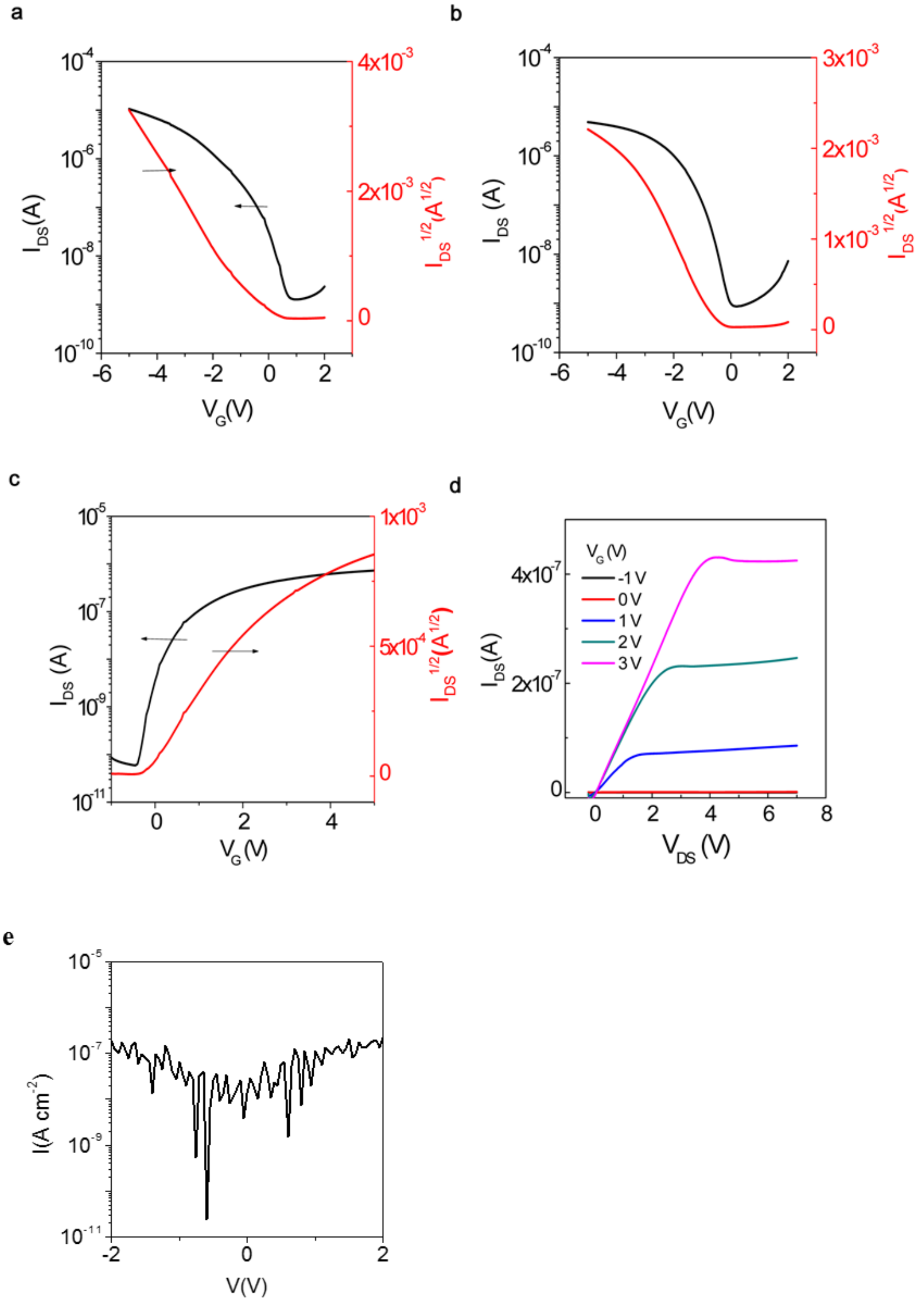

Figure S5. Transfer characteristics of OTFTs from various organic semiconductors on e-PVDF-HFP (1.4 µm thickness). All devices are with bottom-gate and top contact Au drain and source electrodes with L=50 µm, W=1000 µm. All devices were measured in a N_2 atmosphere. **(a)** P3HT (V_{DS} = -5 V), **(b)** PII2T (V_{DS} = -10 V) and **(c)** PCBM (V_{DS} = +7 V). **(d)** output characteristics of PCBM devices made on the e-PVDF-HFP dielectric. **(e)** The curve of gate leakage current vs. voltage measured in an Au/e-PVDF-HFP/Al device with a 1.4-µm-thick e-PVDF-HFP layer.

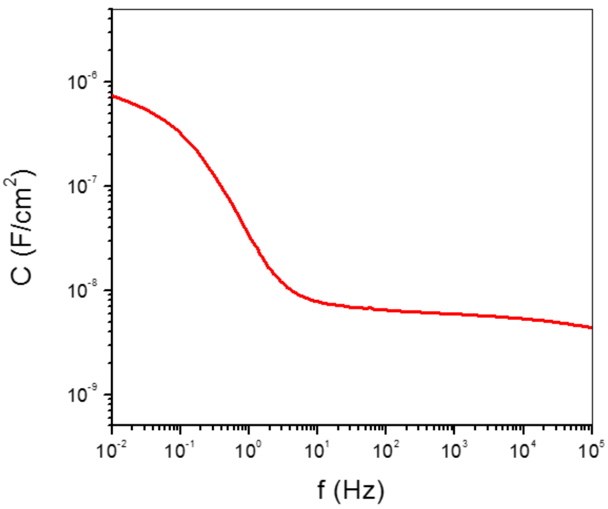

Figure S6. The capacitance of 1.4 μm thick e-PVDF-HFP at different frequencies. The capacitance was measured using a Biologic VMP3 electrochemistry workstation. It is roughly a constant value in the frequency range from 10 Hz to 10^5 Hz, corresponding to a regular charging process of the capacitor. The sharp rise of the capacitance values below 10 Hz is due to electric double layer charging.

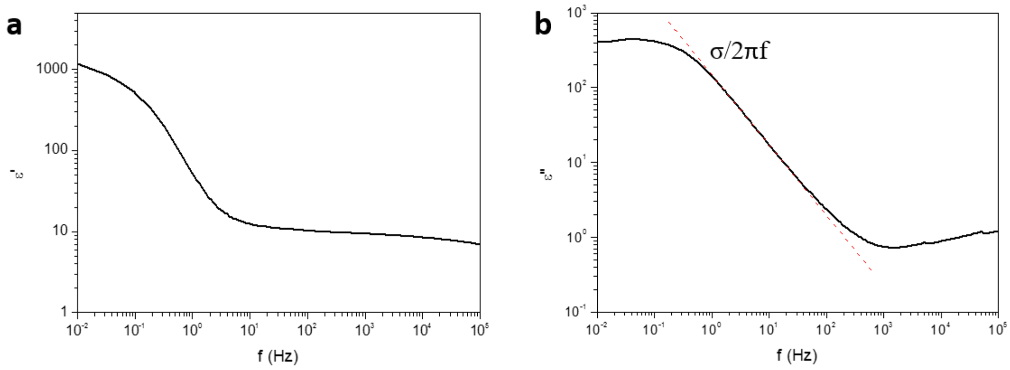

Figure S7. Dielectric permittivity spectra over a wide range of frequencies from 10^{-2} to 10^{5} Hz. ε' **(a)** and ε'' **(b)** denote the real and the imaginary part of the permittivity, respectively. ε'' is inversely proportional to the frequency from ~1 to 100 Hz, which allows the ionic conductivity to be extracted as 8×10^{-11} S/cm. They were measured using a Biologic VMP3 electrochemistry workstation.

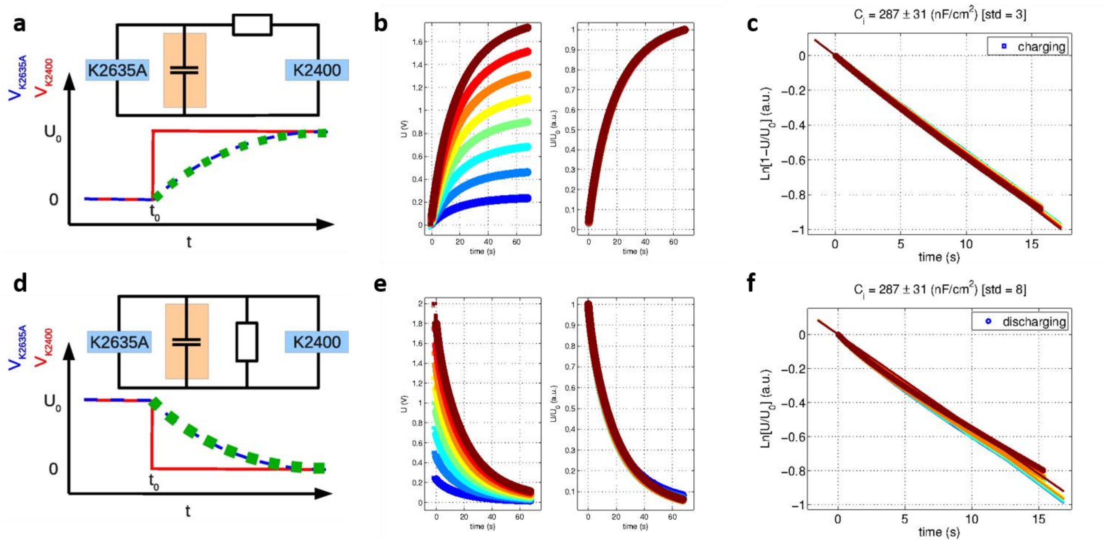

Figure S8. Quasi-DC capacitance measurements based on charging/discharging of an RC circuit. (a) & **(d)** Schematic illustrations of the measurement setups. A Keithley SourceMeter model 2400 was used to charge the circuit. A Keithley SourceMeter model 2635A was used as a voltmeter. For the charging cycle, an electrical resistance of 500 ± 50 MΩ was connected in series with the K2400, while for the discharging cycle the resistor was connected in parallel. **(b)** & **(e)** measured voltage drop on the capacitance sample with applied voltages U_0 = 0.25, 0.5, 0.75, 1, 1.25, 1.5, 1.75 and 2 V. **(c)** & **(f)** calculated gate-capacitance for charging and discharging of a typical device based on e-PVDF-HFP with a thickness of about 1.4 μm exhibiting Ci = 287 ± 31 nF/cm^2.

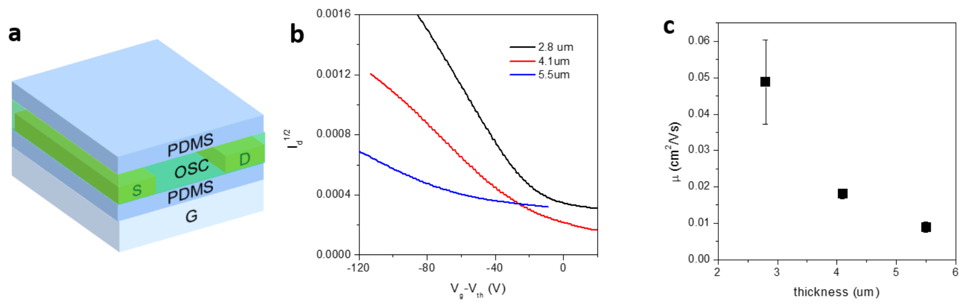

Figure S9. Device characteristics of OTFTs fabricated on PDMS dielectric layers with different thicknesses. (a) Schematic of the transistor device structure. The device adopts a "bottom contact, bottom gate structure". The source/drain electrodes made of Cr/Au (5 nm/40 nm) are pre-patterned on the PDMS dielectric layers of varying thicknesses, followed by a transfer process to laminate the semiconductor onto the electrodes with the assistance of a 5 mm thick PDMS substrate. This process is used because of the swelling issues with PDMS when an organic semiconductor is directly spun on it. **(b)** Transfer curves of OTFTs as a function of the thickness of the PDMS dielectric layer. **(c)** The mobility decreases as the thickness of the PDMS increases. Such a trend has been observed previously in OTFTs employing a low k dielectric material. [Ref: J. Appl. Phys. 105, 034508 (2009).] The higher mobility observed in devices with thinner dielectric layer is ascribed to the effective filling of traps at high charge carrier density.

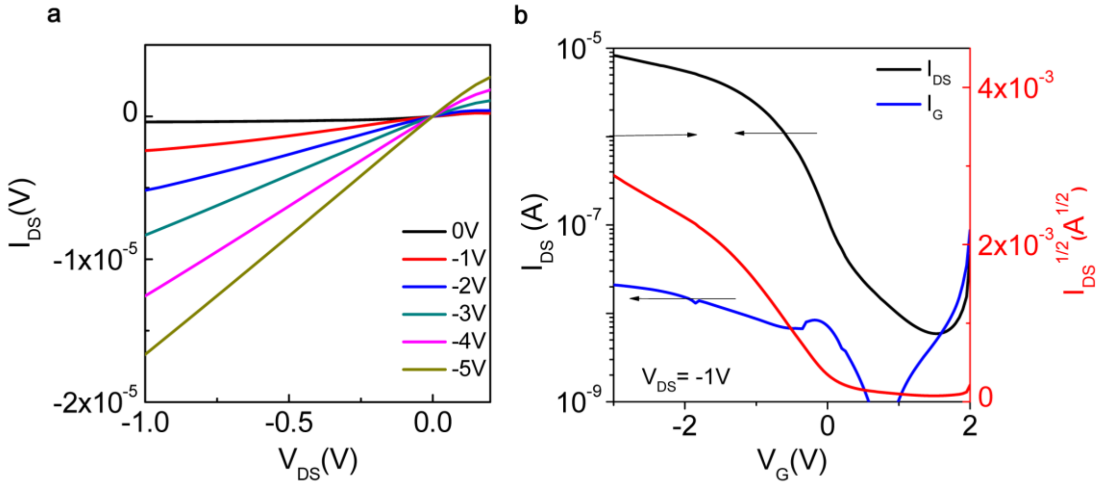

Figure S10 | Linear-regime IV curves of PTDPPTFT4. (a) Output and **(b)** transfer characteristics, of PTDPPTFT4, where channel length = 100 μm and channel width = 2000 μm.

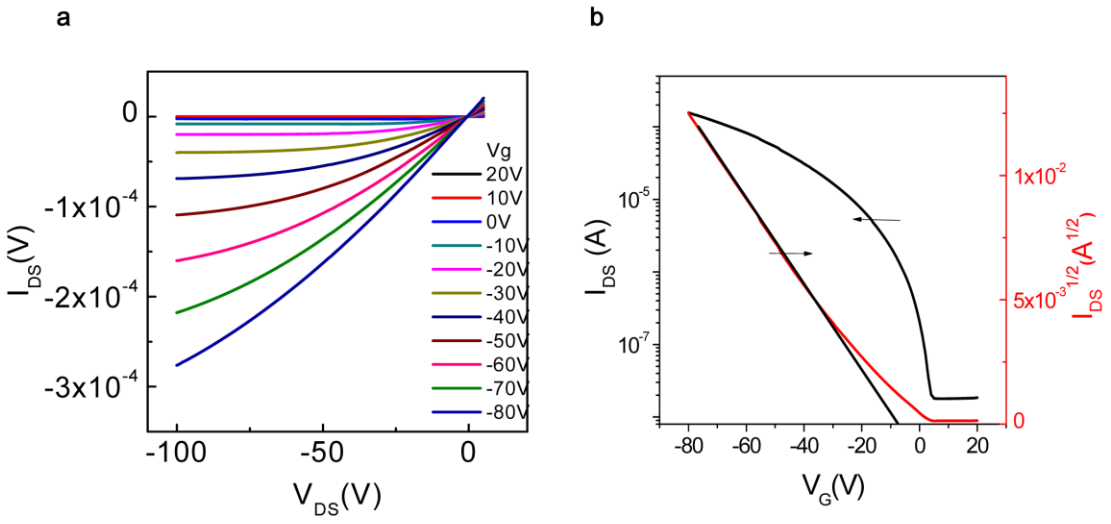

Figure S11. Typical I-V curves of PTDPPTFT4 control devices made on the crystalline PVDF-HFP dielectric layer. (a) Output and **(b)** transfer characteristics (V_{DS} = -80V), where channel length = 50 μm and channel width = 1000 μm capacitance of semicrystalline PVDF-HFP (1.5 μm in thickness) was measured in an inert atmosphere. A high gate voltage of -60 V was needed for a comparable on-current to that of the device fabricated on e-PVDF-HFP (1.4 μm) (10^{-4} A). The calculated mobility from the saturation regime was 0.38 ±0.03 cm^2 V^{-1} s^{-1}. The I_{ON}/I_{OFF} and V_{TH} were 3 ×10^5 and 0 ±4 V, respectively.

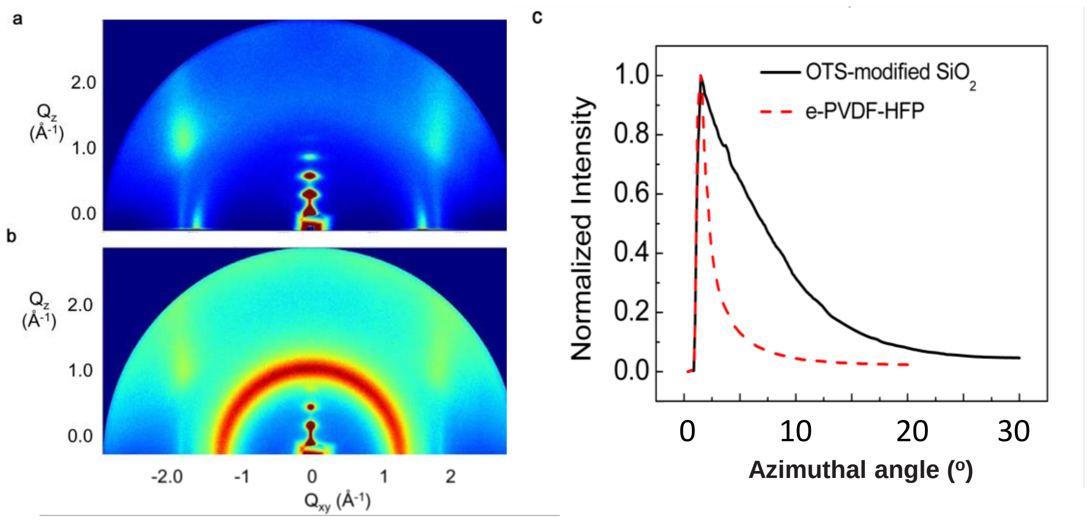

Figure S12 Molecular packing of PTDPPTFT4 thin films on various dielectric surfaces. (a) GIXD pattern of PTDPPTFT4 on the OTS-modified SiO_2 dielectric layer. **(b)** GIXD pattern of PTDPPTFT4 on the ePVDF-HFP. **(c)** Azimuthal XRD profiles at the Q_z = (200) diffraction peak of a PTDPPTFT4 thin film on OTS-modified SiO_2 and ePVDF-HFP, respectively. Compared to films on OTS-modified SiO_2, the PTDPPTFT4 films on e-PVDF-HFP presented a much narrower diffraction peak, which indicates that the polymer semiconductor possessed a larger crystalline size on the surface of e-PVDF-HFP. Additionally, we have found that the π-π stacking distance is reduced from 3.695Å to 3.660 Å on e-PVDF-HFP, and the lamella stacking distance increased from 25.510 Å to 26.658 Å.

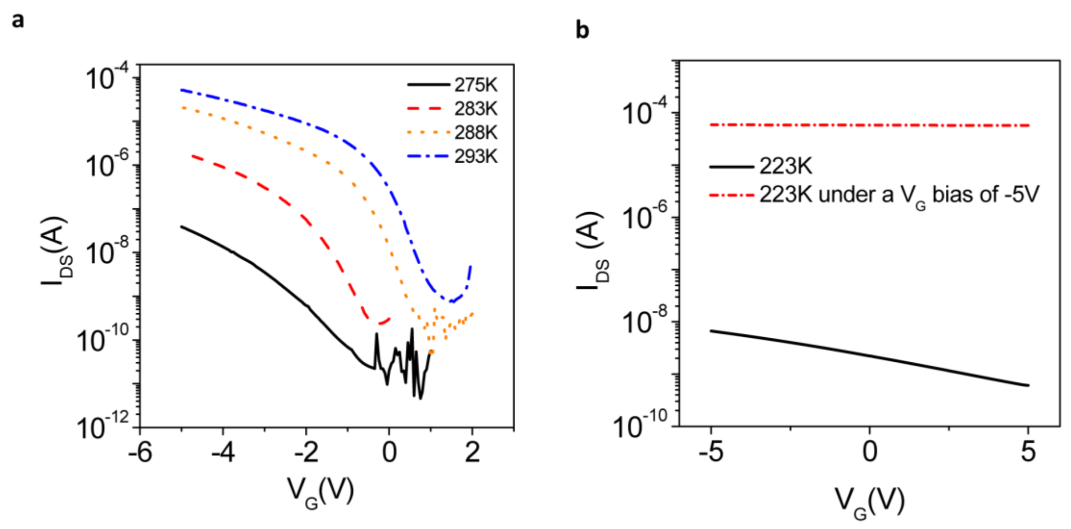

Figure S13 Temperature dependence behavior of PTDPPTFT4 transistors on e-PVDF-HFP dielectric layer (1.4 μm). The TFTs are with bottom gate and top contact Au electrodes with L= 50 μm and W = 1000 μm **(a)** Transfer curves of PTDPPTFT4 FETs operated at the different temperatures. **(b)** Transfer curves of PTDPPTFT4 FETs measured with and without a continuously applied gate bias of -5 V during the cooling. Note that by applying a gate bias in the accumulation mode on the device during cooling down resulted in a high OFF current. This may be due to the low ion mobility at low temperature (below T_g of the polymer).

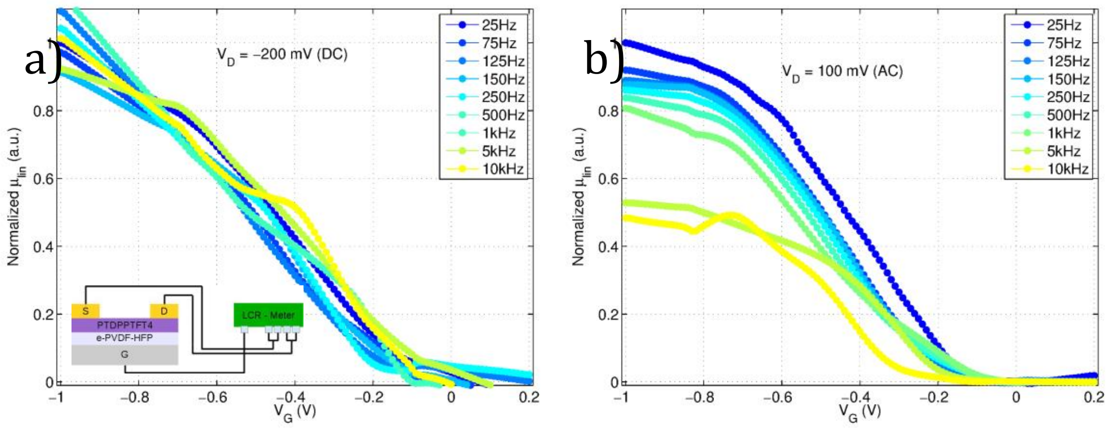

Figure S14 Field-effect mobility frequency dependence of PTDPPTFT4 transistors on e-PVDF-HFP dielectric layer (1.4 μm). The TFTs are with bottom gate and top contact Au electrodes with L= 200 μm**.** Field-effect mobility has been extracted in the linear regime with the quasi-static capacitance. **(a)** Mobility extracted in the linear regime applying a DC voltage of V_D = -200 mV. **(b)** Mobility extracted in the linear regime applying an AC voltage of V_D = 100 mV. The inset shows the measurement setup.

Table S1: Bias stress in organic field effect transistors with different active materials and dielectrics: Threshold voltage shift Δ $V_{TH;}$ threshold voltage shift normalized by bias gate voltage **Δ V_{TH} / V_G bias**; I_D on current of devices at given V_D and V_G; cumulative bias time; polytriarylamine (PTAA), hexa-methyldisilazane (HMDS), poly(4-methoxystyrene) (PMOS), poly(4-methylstyrene) (PMS), and poly(pentafluorostyrene) (PFS).

Materials	Δ V_{TH}	Δ V_{TH} / V_G bias	I_D	Bias V_G	Bias V_D	Bias – time (cumulative time)	Measurement environment	REF
**PTAA based OFETs:**<br>SiO_2/PTAA<br>SiO_2/HDMS/PTAA	- 18 V	0.9	0.15 uA<br>@ V_G = -35 V<br>and V_D = -1V	-20 V	-2V	2 weeks	vacuum:<br>P=10^{-5} mbar	1
**pentacene OFETs:**								
PMOS/Pentacene	– 13.14 V	0.22	20 uA	-60 V	0 V	12 h	Nitrogen	2
PMS/Pentacene	– 9.28 V	0.15	@ V_G = -40 V					
PFS/Pentacene	– 5.05 V	0.08	and V_D = -40V					
**Low voltage pentacene OFETs:**<br>AlOx/Octadecylphosphonic acid/Pentacene *	- 0.3 V	0.1	1 uA<br>@ V_G = -3 V<br>and V_D = -1.5 V	−3 V	−3 V	24 h	Ambient air	3
**Our results:****<br>ePVDF-HFP/PTDPPTFT4	+- 25 mV	0.05	0.5 uA<br>@ V_G =-0.5V	-0.5 V	-0.5 V	120 h	air	this

			and V_D = -0.5 V					work
	+- 25 mV	0.05	0.3 uA @ V_G =-0.5V and V_D = -0.5 V	-0.5 V	-0.5 V	90 h	Di-water	this work

* Degradation of mobility: During the 24-hour bias stress the mobility decreased from 0.6 cm^2 /V s to 0.4 cm^2 /V s *i.e.* 1.4 %/ hour.

** Degradation of mobility only 0.22 % / hour and 0.25% / hour, for measurements in air and DI-water, respectively.

References:

1) Mathijssen, S. G. J. et al. Dynamics of Threshold Voltage Shifts in Organic and Amorphous Silicon Field-Effect Transistors. *Adv. Mater.*, **19,** 2785, DOI: 10.1002/adma.200602798 (2007)
2) Kim, J. et al. The Origin of Excellent Gate-Bias Stress Stability in Organic Field-Effect Transistors Employing Fluorinated-Polymer Gate Dielectrics. *Adv. Mater.* **26,** 7241, DOI: 10.1002/adma.201402363 (2014),
3) Zschieschang, U., Weitz, R. T. & Kern, K. Bias stress effect in low-voltage organic thin-film transistors. *App. Phys. A*, **95,** 139, DOI 10.1007/s00339-008-5019-8 (2009),
